# *Helicobacter pylori* Colonization Ameliorates Glucose Homeostasis in Mice through a PPAR γ-Dependent Mechanism

**DOI:** 10.1371/journal.pone.0050069

**Published:** 2012-11-15

**Authors:** Josep Bassaganya-Riera, Maria Gloria Dominguez-Bello, Barbara Kronsteiner, Adria Carbo, Pinyi Lu, Monica Viladomiu, Mireia Pedragosa, Xiaoying Zhang, Bruno W. Sobral, Shrinivasrao P. Mane, Saroj K. Mohapatra, William T. Horne, Amir J. Guri, Michael Groeschl, Gabriela Lopez-Velasco, Raquel Hontecillas

**Affiliations:** 1 Nutritional Immunology and Molecular Medicine Laboratory, Center for Modeling Immunity to Enteric Pathogens, Virginia Bioinformatics Institute, Virginia Tech, Blacksburg, Virginia, United States of America; 2 Department of Biology, University of Puerto Rico, Río Piedras. San Juan, Puerto Rico, United States of America; 3 Erlangen University, Erlangen, Germany; 4 Department of Biomedical Sciences and Pathobiology, VA-MD Regional College of Veterinary Medicine, Virginia Tech, Blacksburg, Virginia, United States of America; Institut Pasteur Paris, France

## Abstract

**Background:**

There is an inverse secular trend between the incidence of obesity and gastric colonization with *Helicobacter pylori*, a bacterium that can affect the secretion of gastric hormones that relate to energy homeostasis. *H. pylori* strains that carry the *cag* pathogenicity island (*PAI*) interact more intimately with gastric epithelial cells and trigger more extensive host responses than *cag^−^* strains. We hypothesized that gastric colonization with *H. pylori* strains differing in *cag PAI* status exert distinct effects on metabolic and inflammatory phenotypes.

**Methodology/Principal Findings:**

To test this hypothesis, we examined metabolic and inflammatory markers in db/db mice and mice with diet-induced obesity experimentally infected with isogenic forms of *H. pylori* strain 26695: the *cag PAI* wild-type and its *cag PAI* mutant strain 99–305. *H. pylori* colonization decreased fasting blood glucose levels, increased levels of leptin, improved glucose tolerance, and suppressed weight gain. A response found in both wild-type and mutant *H. pylori* strain-infected mice included decreased white adipose tissue macrophages (ATM) and increased adipose tissue regulatory T cells (Treg) cells. Gene expression analyses demonstrated upregulation of gastric PPAR γ-responsive genes (i.e., CD36 and FABP4) in *H. pylori*-infected mice. The loss of PPAR γ in immune and epithelial cells in mice impaired the ability of *H. pylori* to favorably modulate glucose homeostasis and ATM infiltration during high fat feeding.

**Conclusions/Significance:**

Gastric infection with some commensal strains of *H. pylori* ameliorates glucose homeostasis in mice through a PPAR γ-dependent mechanism and modulates macrophage and Treg cell infiltration into the abdominal white adipose tissue.

## Introduction

*Helicobacter pylori* is the dominant member of the gastric microbiota and has persistently colonized the stomach in humans since our early evolution [1]. However, currently in developed countries there has been a sharp decrease in the prevalence of *H. pylori* gastric colonization [2], [3], [4]. Colonization with strains bearing the *cag* (cytotoxin-associated gene) pathogenicity island (*cag PAI*) is associated with increased risk of distal gastric pathologies such as non-cardia gastric adenocarcinoma, gastric lymphoma and peptic ulceration [5], [6]. Conversely, there also is increasing evidence of *H. pylori* protection against esophageal and cardial pathologies [2], [7], [8], [9], childhood asthma [10], [11], [12] and childhood allergies [11], [13]. The mechanisms underlying this protective effect of *H. pylori* acting as a commensal bacterium are largely unknown, although for asthma the suppression of T helper 2 responses by a neutrophil-activating protein of *H. pylori* favorably modulates allergic asthma in mice [14].

**Table 1:** Effect of *Helicobacter pylori* infection of db/db mice on gastric leptin mRNA expression and on plasma hormonal concentrations on day 71 post-challenge^1^.

Hormone	*H. pylori* 98–325 *cag* PAI WT	*H. pylori* 99–305 *cag* PAI mutant	Uninfected (control)
Gastric leptin (SQ^2^ cDNA x 10^14^)	2.9±0.1*	14.3±0.9*	0.009±0.0007
Plasma leptin (ng/mL)	73.4±9.5*	62.4±12.2*	41.6±12.2
Plasma ghrelin (pg/mL)	1,945.8±197.2	1,789.6±200.2	2,077.6±254.4
Plasma insulin (ng/mL)	2.6±0.9	4.8±1.1	1.8±1.2

1 Statistically significant differences (*P*<0.05) in comparison to the non-colonized control (*) are indicated (n = 10 mice/group).

2 Starting quantity (SQ) of cDNA per microgram of gastric RNA.

**Table 2:** Effect of *Helicobacter pylori* infection of C57BL6/J mice with diet-induced obesity on gastric interleukin-6 and leptin mRNA expression on day 60 post-challenge^1^.

Hormone	*H. pylori* 99–305 *cag* PAI mutant	Uninfected (control)
Gastric IL-6 (SQ^2^ cDNA ×10^5^)	6.0±0.4*	10.0±0.8
Gastric leptin (SQ cDNA ×10^5^)	12.0±0.9*	3.0±0.1

1 Statistically significant differences (*P*<0.05) in comparison to the non-colonized control (*) are indicated (n = 10 mice/group).

2 Starting quantity (SQ) of cDNA per microgram of gastric RNA.

The *cag PAI* encodes a type IV secretion system that mediates interactions between the bacterium and the gastric epithelium [15], including the secretion of the *cag* effector protein (CagA) and peptidoglycan. *H. pylori* virulence factors, such as CagA and the vacuolating protein (VacA), mediate the interactions of *H. pylori* with host cells [16], [17], although immune modulation mediated by *H. pylori* also may utilize other pathways [18], [19]. Transgenic expression of CagA in mice leads to gastric hyperplasia by causing aberrant epithelial cell signaling [20], [21]. Inside the host cell, phosphorylation of tyrosines within CagA EPIYA repeats [22] induces the hummingbird phenotype [23], whereas CRPIA motifs (*c*onserved *r*epeat responsible for *p*hosphorylation-*i*ndependent *a*ctivity) contribute to the epithelial proliferative and pro-inflammatory responses [24]. CagA proteins lacking EPIYA motifs induce the Jak/STAT3 pathway, with effects on c-*myc* [25]. CagA alters tight junction barrier function in polarized epithelial cells, affecting adhesion and basement membrane integrity [26], [27], and exposing the gastric lamina propria to luminal antigens. A newly characterized strain of *H. pylori* (V225d) contains an atypical but active *cag PAI*; passage through mice led to loss of host cell interactive phenotypes *via* a 15-kb deletion within the *cag PAI* [1]. Thus, the deletion of a significant portion of the *cag* PAI in *H. pylori* resulted in suppressed host cell interactive phenotypes. In addition to illustrating the value of comparing effects of *cag* PAI-positive and –negative strains on cell phenotype, these findings suggest the potential importance of *H. pylori cag* PAI in regulating immunity and metabolism.

**Figure 1:**
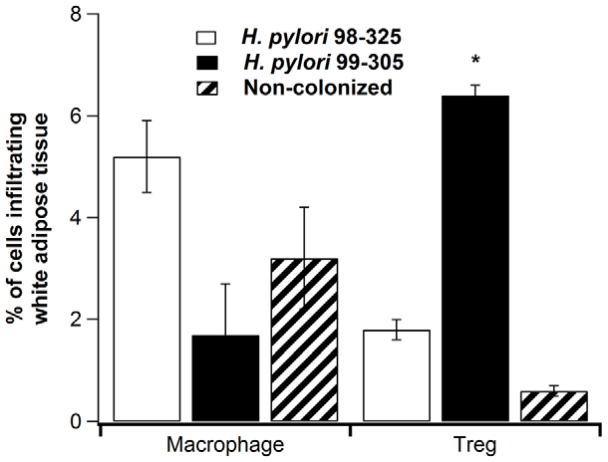
Effect of *Helicobacter pylori* infection on infiltration of immune cell subsets into adipose tissue. Macrophages (F4/80^+^CD11b^+^) and regulatory T cells (CD4^+^CD25^+^Foxp3^+^) were immunophenotyped in white adipose tissue (WAT) from leptin receptor-deficient (db/db) mice infected with either the wild-type *H. pylori* 98–325 (white bars), the isogenic *H. pylori* 99–305 (black bars), or uninfected (dashed bars), (n = 10 mice/group). Statistically significant differences (*P*<0.05) between treatments (*) are indicated.

In addition to its interaction with the gastric epithelium, *H. pylori* also interacts with gastric neuroendocrine cells secreting gastrin, somatostatin, leptin, and ghrelin, and may influence metabolic processes. Specifically, gastric *H. pylori* colonization decreases plasma levels of ghrelin, a hormone involved in energy homeostasis [28], [29] as well as the density of gastric ghrelin-producing cells in obese patients [30]. Plasma ghrelin concentrations increase following *H. pylori* eradication, suggesting that eradication may contribute to increased appetite and weight gain, and potentially affect body mass index [29]. Ghrelin expression is negatively regulated by leptin, a multifunctional adipokine with cytokine-like features [31]. Leptin is chiefly synthesized by adipocytes, but 5 to 10% is produced in the stomach [32], [33], [34], [35]. Plasma leptin concentrations are not fully dependent on adiposity [35], suggesting a contribution of gastric-derived leptin.

**Figure 2:**
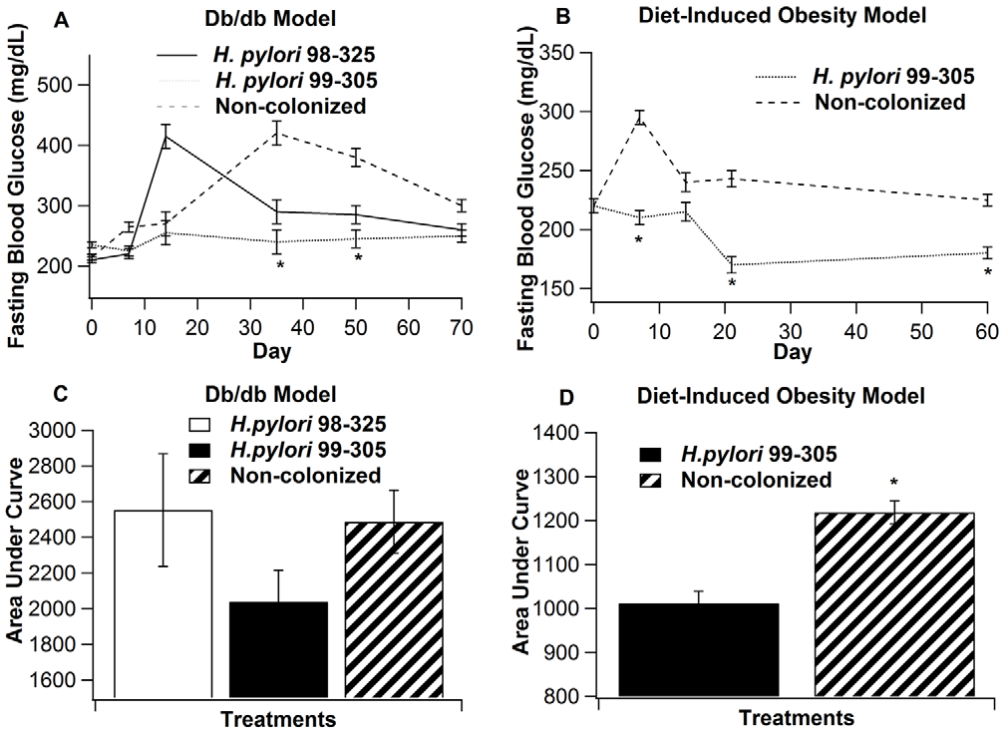
Effect of *Helicobacter pylori* infection on fasting blood glucose concentrations in two murine models of obesity. **Panel** **A**: Fasting blood glucose (FBG) concentrations from leptin receptor-deficient (db/db) mice infected with either the wild-type *H. pylori* 98–325 (solid line), the isogenic *H. pylori* 99–305 (dotted line), or uninfected (dashed line), (n = 10 mice/group). Blood was obtained on days 0, 7, 14, 35, 50 and 71 of the study. **Panel B**: FBG concentrations in a mouse model of diet-induced obesity (DIO). Uninfected (control) mice (dashed line) or mice infected with *H. pylori* 99–305 (dotted line) are shown. Blood was obtained on days 0, 7, 14, 21 and 60 of the study. **Panels C & D** illustrate the area under the curve calculations for FBG concentrations in the db/db and DIO models, respectively. Statistically significant (*P*<0.05) differences with the uninfected control mice are indicated (*), (n = 10 mice/group).

Obesity is increasing in both developed and developing countries [36], and the incidence of type II diabetes has grown concomitantly [37]. This global epidemic coincides with the decreasing prevalence of *H. pylori* [2], [3], [38], [39], [40], suggesting that gastric colonization with this bacterium might contribute to anti-obesity and anti-diabetic actions. Obesity is characterized by insulin resistance and low-grade chronic inflammation in white adipose tissue (WAT) with accumulation of macrophages and elevated levels of circulating pro-inflammatory cytokines [41]. Macrophages mediate chronic inflammation in WAT, and are implicated in obesity-induced inflammation and insulin resistance [42], [43]. In the gastric mucosa, *H. pylori* is pro-inflammatory [44], [45], but down-modulates the immune response by impairing phagocytosis [46] and enhancing apoptosis of macrophages [47]. By targeting cells involved in the immune response, *H. pylori* enhances its own persistence in the host [14], [48], suggesting possible global roles for *H. pylori* in the induction of anti-inflammatory or regulatory responses. Since *cag* ^+^ *H. pylori* strains trigger stronger inflammatory responses than do *cag* ^−^ strains [1], we hypothesized that *cag* status could affect energy homeostasis through its neuroendocrine and immunological effects. To test this hypothesis, we examined the effects of gastric *H. pylori* infection on appetite-controlling hormones and peroxisome proliferator-activated receptor γ (PPAR γ), a nuclear receptor and transcription factor that acts as an important thermostat for inflammation and metabolism. We used two mouse models of obesity-related inflammation (i.e., leptin receptor deficient *db/db* mice and mice with diet-induced obesity, DIO). Leptin regulates immune responses by direct effects on immune cells and regulates feeding and the neuroendocrine system by acting on its receptor in the hypothalamus [49]. The *db/db* mouse model lacks the long isoform of the leptin receptor (ObRb), which associates with the Janus kinase 2 to mediate intracellular signaling. This mutation causes hyperphagia and decreased metabolic rate, predisposition to diabetes, and endocrine dysregulation. The DIO mouse model consists on feeding high-fat diets (40 calories from fat) to induce obesity and white adipose tissue inflammation [50]. By using these mouse models of obesity and diabetes in combination with *H. pylori* infection, we examined the role of the predominant gastric bacterium in regulating the initiation, progression and outcomes of obesity and metabolic disorders.

**Figure 3:**
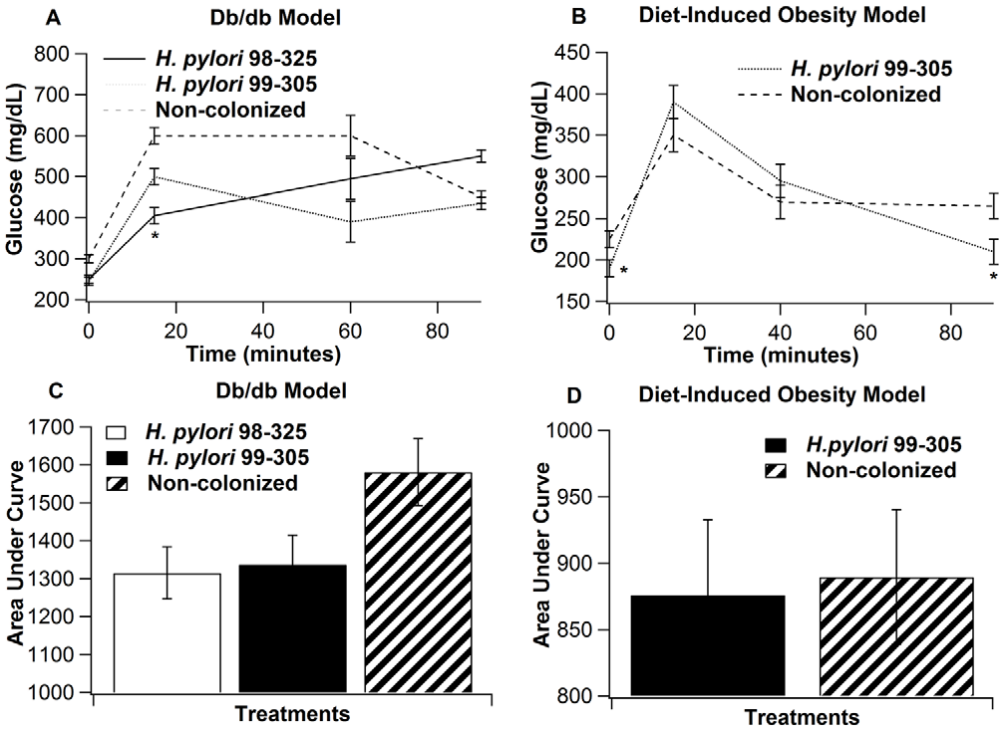
Effect of *Helicobacter pylori* infection on plasma glucose concentrations, obtained from a glucose tolerance test (GTT). Mice were administered an intraperitoneal glucose challenge (2 g/kg body weight). **Panel A**: Glucose levels in leptin receptor-deficient (db/db) mice infected with either the wild-type *H. pylori* 98–325 (solid line), the isogenic *H. pylori* 99–305 (dotted line), or uninfected (control) (dashed line). Blood was collected before (0), then 15, 60, and 90 minutes after glucose load, (n = 10 mice/group). **Panel B**: Mouse model of diet-induced obesity; DIO mice infected with *H. pylori* 99–305 (dotted line), or uninfected (dashed line). **Panels C & D** illustrate the area under the curve calculations for the glucose concentrations during a GTT in the db/db and DIO models, as in Panels A & B respectively. Blood was collected before (0), then 15, 45, and 90 minutes of glucose load, (n = 10 mice/group). Statistically significant differences (*P*<0.05) between treatments (*) are indicated.

**Figure 4:**
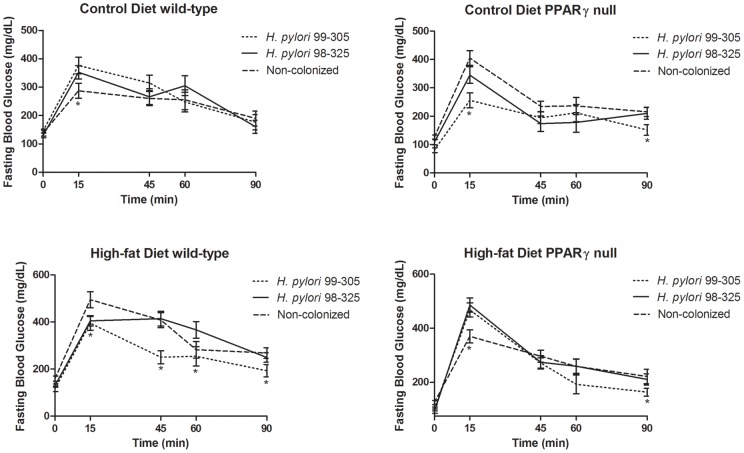
Effect of *Helicobacter pylori* infection on plasma glucose concentrations in wild-type and peroxisome proliferator-activated receptor (PPAR) γ null mice. Mice were administered an intraperitoneal glucose challenge (2 g/Kg body weight). Blood was collected before (0), then 15, 45, 60, and 90 minutes after glucose load, (n = 10 mice/group). **Panel A and B:** Wild-type and PPAR γ null mice fed regular AIN-93G diets infected with either *H. pylori* 99–305 or 98–325, when compared to the uninfected group. **Panel C and D:** Wild-type and PPAR γ null mice fed high-fat diets infected with either *H. pylori* 99–305 or 98–325, when compared to the uninfected group. **Panel C:** Plasma glucose levels were significantly lower at 15, 45 and 60 min in wild-type mice, fed high-fat diets infected with *H. pylori* 99–305 when compared to the uninfected or infected with *H. pylori* 98–325. **Panel D:** PPAR γ null mice fed high-fat diets showed significant differences between strains or the uninfected group only at t = 15 min. Statistically significant differences (*P*<0.05) between treatments (*) are indicated.

## Materials and Methods

### Diets and mouse model systems

BKS.Cg−+Leprdb/+Leprdb/OlaHsd(db/db) mice, which lack the long isoform of the leptin receptor (ObRb), were fed purified diets that represent a modification of the AIN-93G rodent diet in which the nutritional requirements were met or exceeded (Table S1). We utilized 30 genetically obese db/db pre-diabetic mice in the first experiment. We also performed a follow-up experiment using twenty C57BL/6 wild-type mice in a model of diet-induced obesity (DIO), as described [50]. In the second study we only used the *cag* PAI mutant strain because the results of the first study had revealed more differences with the mutant than the wild-type strain. A third experiment used PPAR γ-expressing (wild-type, PPAR γ fl/fl; MMTV-Cre-) and mice lacking PPAR γ in immune and epithelial cells (PPAR γ fl/fl; MMTV−Cre+) in a C57BL/6 background. These mice were fed either control AIN-93G or high-fat diets (Table S1). Mice were either uninfected or challenged with *cag PAI*+ (wild-type) and isogenic *cag PAI*-mutant strains of *H. pylori* and a group of control non-colonized mice (n = 10/group) as described below. All experimental procedures were approved by the Institutional Animal Care and Use Committee (IACUC) of Virginia Tech and met or exceeded requirements of the Public Health Service/National Institutes of Health and the Animal Welfare Act.

**Figure 5:**
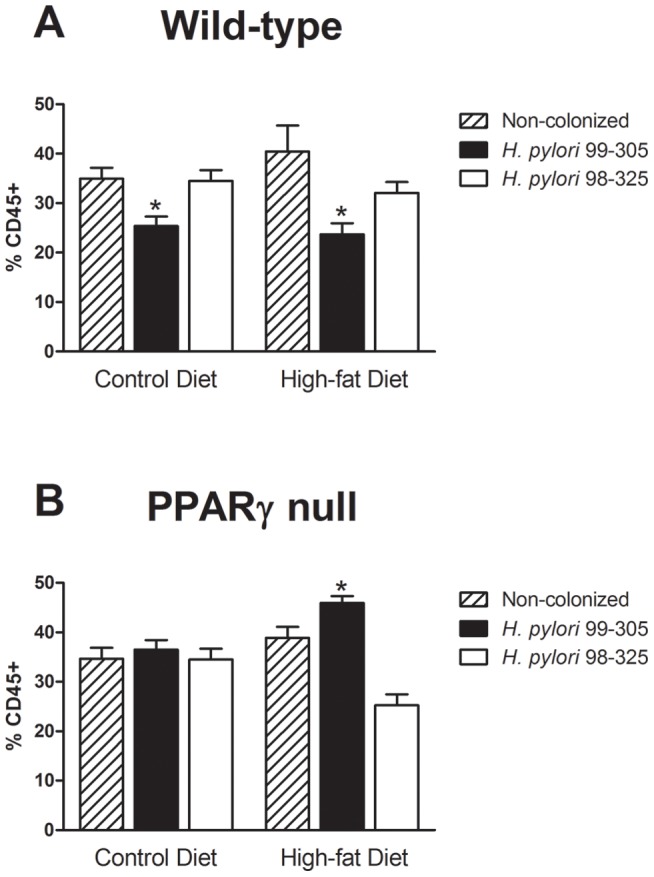
Effect of *Helicobacter pylori* infection on white adipose tissue (WAT) macrophage infiltration in wild-type and peroxisome proliferator-activated receptor (PPAR) γ null mice. (**A**) Percentages of F4/80+CD11b+ infiltrating macrophages in WAT in wild-type mice fed high-fat diets, infected with *H. pylori* 99–305, 98–325 or uninfected controls. (**B**) Percentages of F4/80+CD11b+ infiltrating macrophages in WAT in PPAR γ null mice fed high-fat diets, infected with *H. pylori* 99–305, 98–325 or uninfected controls. Statistically significant differences (*P*<0.05) between treatments (*) are indicated.

### Bacterial culture conditions

*Helicobacter pylori* was grown on Columbia blood agar (BD, Sparks, MD) supplemented with 7% lacked horse blood (Lampire biological laboratories, Piperscille, PA) plates. Cultures were incubated at 37°C for 4 days in an anaerobic jar (OXOID, UK) containing a campylobacter system for the generation of microaerophilic conditions composed of 5% oxygen, 10% carbon dioxide and 85% nitrogen.

**Figure 6:**
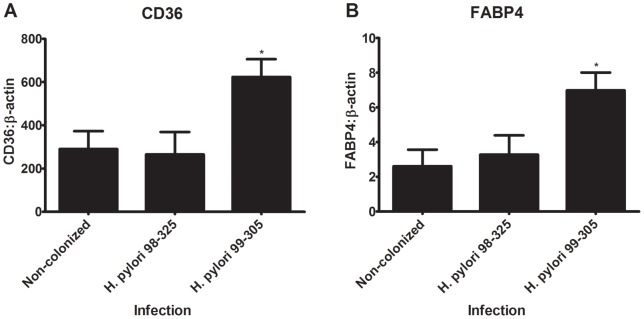
Effect of *Helicobacter pylori* infection on peroxisome proliferator receptor (PPAR) γ responsive gene expression. Gastric expression of CD36 (A) and fatty acid binding protein (FABP4) (B) was assessed by real-time quantitative RT-PCR in wild type mice fed high-fat diet infected with *H. pylori* 98–325 or 99–305 strains, or uninfected controls (n = 10). Data are represented as mean ± standard error. Points with an asterisk are significantly different when compared to the wild type control group (*P*<0.05).

### Experimental *H. pylori* infection

Eight-week-old mice were challenged with *cag PAI*+ (wild-type) and isogenic *cag PAI*-mutant strains of *H. pylori* and a group of control non-colonized mice (n = 10/group). Specifically, we used strain 98–325 (wild-type, mouse-passed *cag PAI*+ European 26695) and strain 99–305 which has a knockout of the entire *cag PAI* made by transformation of 98–325 with a PCR product from a strain in which the chloramphenicol resistance cassette replaced the entire *cag PAI* [51]. The absence of *cagA* in the *cag PAI* mutant strain 99–305, and its expression in wild-type strain 98–325, as well as the presence of *vacA* in both strains, was confirmed by PCR (data not shown). *H. pylori* gastric infection with the strains was carried out by orogastric gavage. Briefly, freshly prepared aliquots (10^10^ colony forming units) of the *H. pylori* strains in sterile Brucella broth were administered to mice three times on days 1, 3, and 5 of the study by oral gavage needle. This dose was used in all experiments. All uninfected mice (n = 10) were inoculated with the same volume of sterile Brucella broth alone. To increase the pH of the stomach and facilitate bacterial colonization, mice were treated with 5% Urea on the drinking water for 7 days starting the day of the first infection.

### Assessment of body weight and glucose tolerance

All thirty mice were determined to be normoglycemic (fasting blood glucose levels lower than 250 mg/dl) and to have similar weights (20±1.5 g) prior to the experimental challenge with *H. pylori*. Mice were weighed on a weekly basis and examined for clinical signs of disease by blinded observers. After a standard 12 h fast, glucose was determined on days 0, 7, 14, 35, 50, and 71 of the study for db/db mice and on days 0, 7, 14, 21, and 60 for mice with DIO. Briefly, blood was collected via the lateral tail vein and placed onto capillary blood collection tubes. Mice then were administered a glucose tolerance test by intraperitoneal injection of D-glucose (2 g/kg body weight) and blood samples collected prior to the injection (time 0) (corresponding to a baseline FBG level following a 12-h fast starting at 6 a.m.) and at 15, 60, and 90 minutes (db/db model) or 15, 45 and 90 minutes (DIO model) following the glucose injection.

### Necropsy procedures

Mice were euthanized by CO_2_ narcosis with secondary thoracotomy on day 71 of the study (db/db model) or day 60 of the study (DIO model). Blood was collected from the heart in heparinized tubes and centrifuged for 10 minutes at 5000 rpm. Plasma was collected and stored at −80°C for ELISA and RIA analyses. The abdominal white adipose tissue (WAT) and interscapular brown adipose tissue (BAT) were excised and weighed. Stomach samples were collected for detection of *H. pylori* in the gastric mucosa by PCR. Omental adipose tissue and gastric tissue samples also were collected in RNA-later (Ambion, Austin, TX) for RNA isolation and gene expression analyses.

### Assessment of plasma leptin, insulin and ghrelin concentrations

Assays were performed for mouse-specific leptin [ELISA; (Linco)], insulin [ELISA, (Linco)] and ghrelin [RIA (Mediagnost)]. Each assay was performed according to the manufacturer's instructions.

### White adipose tissue fractionation

WAT was fractionated into stromal vascular cells (SVCs) and adipocytes, as described [50], [52]. Briefly, WAT was excised, weighed, minced into small (<10 mg) pieces and placed into digestion media consisting of DMEM (Mediatech, Herndon VA) supplemented with 2.5% HEPES (Mediatech) and 10 mg/mL fatty-acid free bovine serum albumin (FAB-poor BSA, Sigma), Liberase Blendzyme 3 (0.03 mg/mL, Roche) and DNase I (50 U/mL, Qiagen, Valencia CA). Samples were incubated in a rotating 37°C water bath for 90 min, filtered through a 250 μm nylon mesh (Sefar America Inc., Depew NY) to remove undigested particles, and centrifuged at 4°C at 1,000 x g for 10 min. The pellet, consisting of SVCs, containing endothelial cells, pre-adipocytes, macrophages and T cells, was washed with DMEM and centrifuged at 4°C at 1,000 x g for 10 min. The supernatant was discarded and erythrocytes lysed by incubating the SVCs in 2 mL erythrocyte lysis buffer for 2 min before stopping the reaction with 9 mL phosphate-buffered saline (PBS). Cells then were re-centrifuged at 4°C at 1,000 x g for 10 min, suspended in 1 ml of PBS, and enumerated with a Z1 Single Particle Counter (Beckman Coulter, Fullerton CA). The SVCs were resuspended in FACS buffer (PBS, 1% normal goat serum, 0.2% sodium azide) at a concentration of 2×10^6^ cells/mL.

### Flow cytometry

WAT-derived SVCs (2×10^5^ cells) were seeded into 96-well plates, centrifuged at 4°C at 1,800 x g for 4 min, then incubated in the dark at 4°C for 20 min in FcBlock (20 μg/ml; BD Pharmingen), and then for 20 min with fluorochrome-conjugated primary antibodies anti-F4/80-PE-Cy5 (5 μg/mL), anti-CD11b-FITC (2 μg/mL) (eBioscience), anti-CCR2-PE (R&D systems, Minneapolis MN). The specific antibody combinations used for assessing macrophage infiltration into WAT were F4/80, CD11b, CCR2. For assessing regulatory T cell infiltration into WAT we used the following combinations: anti-CD4-FITC and anti-CD25-Pe-Cy5 (BD Pharmingen), anti-Foxp3-PE (1 μg/mL) (eBioscience). After incubation with primary antibodies, cells were centrifuged at 4°C at 1,800 x g for 4 min and washed with 200 μL of FACS buffer. After washing, cells were suspended in 200 μL PBS and 3-color data acquisition performed on a FACS Calibur flow cytometer. Data analyses were performed using the CellQuest software (BD).

### Quantitative real-time reverse transcriptase PCR

Total RNA was isolated from stomachs using the RNA isolation Minikit (Qiagen), according to the manufacturer's instructions, including a DNAase digestion step. Total RNA (1 μg) was used to generate complementary DNA (cDNA) template using the iScript cDNA Synthesis Kit (Bio-Rad, Hercules CA). The total reaction volume was 20 μL with the reaction incubated as follows in an MJ MiniCycler: 5 min at 25°C, 30 min at 52°, 5 min at 85°C, hold at 4°C. PCR was performed on the cDNA using Taq DNA polymerase (Invitrogen), as described [53]. Each gene amplicon was purified with the MiniElute PCR Purification Kit (Qiagen) and quantitated on an agarose gel by using a DNA mass ladder (Promega). These purified amplicons were used to optimize real-time PCR conditions and to generate standard curves in the real-time PCR assay. Primer concentrations and annealing temperatures were optimized for the iCycleriQ system (Bio-Rad) for each set of primers using the system's gradient protocol. PCR efficiencies were maintained above 92% and correlation coefficients above 0.98 for each primer set (Table S2) during optimization and also during the real-time PCR of sample DNA.

Complementary DNA (cDNA) concentrations for genes of interest were examined by real-time quantitative PCR using an iCycler IQ System and the iQ SYBR green supermix (Bio-Rad). Standard curves were generated using 10-fold dilutions of purified amplicons starting at 5 pg of cDNA and used later to calculate the starting amount of target cDNA in the unknown samples. SYBR green I is a general double-stranded DNA intercalating dye and may therefore detect nonspecific products and primer/dimers in addition to the amplicon of interest. To determine the number of products synthesized during the real-time PCR, melting curve analysis was performed. Real-time PCR was used to measure the starting amount of nucleic acid of each unknown sample of cDNA on the same 96-well plate.

### Statistics

Data were analyzed by analysis of variance (ANOVA), performed by using the general linear model procedure of SAS [54] as previously described [55], [56]. A probability value (*P*)<0.05 was considered to be significant. Experiments 1 and 2 were analyzed as completely randomized studies, and experiment 3 was analyzed as a (2×2×3) factorial arrangement within a completely randomized design. ANOVA was utilized to determine the main effects of the dietary treatment (low-fat vs. high-fat diet), mouse genotype (wild-type vs. PPAR γ knockout), or the infection status (uninfected, infected with the wild-type strain or infected with the *cag* PAI mutant strain) and the 2-way and 3-way interactions between dietary treatment, mouse genotype, and infection status. When the model was significant, ANOVA was followed by Sheffe's post-hoc multiple comparison method and statistically significant (*P*<0.05) differences among treatment groups are depicted using different superscripts.

## Results

### Gastric gene expression and plasma levels of leptin

We assayed expression of gastric leptin and circulating levels of leptin, ghrelin, and insulin in the db/db mouse model. The real-time qRT-PCR and ELISA results indicate that gastric leptin mRNA and plasma leptin protein concentrations were higher in the *H. pylori*-colonized mice, regardless of the *cag* PAI status, in comparison to the non-colonized group (Table 1). While mice challenged with the *cag PAI* mutant strain 99–305 had lower plasma levels of ghrelin and higher levels of insulin than the other groups, the differences were not statistically significant. Consistent with the findings in the genetically obese mouse model, in DIO mice gastric infection with the *cag PAI* mutant strain resulted in a 4-fold increase in gastric leptin mRNA levels compared to non-colonized mice (Table 2). In addition, gastric infection with the *cag PAI* mutant strain suppressed gastric IL-6 mRNA expression, thus, indicating lower levels of gastric inflammation in mice colonized with the mutant strain.

### Infiltration of macrophages and regulatory T cells into white adipose tissue (WAT)

Adipose tissue macrophages (ATM) are primary contributors to obesity-related inflammation and the derived insulin resistance [41], but their effector function is suppressed by CD4^+^CD25^+^Foxp3^+^ regulatory T cells (Treg) [57]. In addition to its role in metabolism, leptin has been recognized as an important immunoregulatory hormone and both T cells and macrophages express leptin receptors. We first isolated the stromal vascular fraction (SVF) of WAT, containing lymphocytes, macrophages, fibroblasts, endothelial cells, and pre-adipocytes, and then examined the macrophage and lymphocyte subsets within this fraction based on surface expression of glycoproteins F4/80 and CD11b. Mice infected with the *cag PAI* mutant 99–305 had a lower proportion of ATM and greater numbers of Tregs in the SVF compared to uninfected mice, or to mice infected with the *cag*-positive wild-type *H. pylori* strain 98–325 (Figure 1). This study provides evidence that gastric *cag* PAI-negative *H. pylori* colonization affects adipose tissue inflammatory cell populations.

### Effect of *H. pylori* infection on body weight

Body weights of mice infected with the *cag PAI* mutant were consistently lower than mice in the other two groups from day 14 before equalizing by day 70, but differences only were significant on day 42 (data not shown). We further examined the effect of the *cag PAI* mutant strain 99–305 on obesity, by performing a follow-up study using the diet-induced obesity (DIO) mouse model and examining abdominal and subcutaneous fat accumulation. We found that gastric infection with the *cag PAI* mutant dramatically decreased visceral WAT accumulation in comparison to uninfected control mice (0.23 vs 0.46 grams, *P*<0.04).

### Fasting blood glucose (FBG) and insulin concentrations

We determined the effect of *H. pylori* on glucose homeostasis by measuring fasting blood glucose concentrations following experimental infection in two models of obesity (Figure 2). In the db/db model, on days 35 and 50, fasting blood glucose levels were significantly higher in the uninfected mice (n = 10/group) compared to the *H. pylori*-infected mice, regardless of the strain of *H. pylori* utilized, with the lowest FBG concentrations in mice colonized with the *cag PAI* mutant strain 99–305, throughout the course of the study (Figure 2A). We confirmed the effect of infection with the *cag PAI* mutant strain 99–305 using the DIO model (Figure 2B). Area under the curve (AUC) calculations show that mice infected with the *cag PAI* mutant strain 99–305 had the lowest glucose levels in both models (Figure 2C&D). Together, these studies provide evidence of the beneficial effects of colonization with *H. pylori* on glycemic control, at least in the two mouse models used in this study.

### Effect of *H. pylori* infection and PPAR γ expression on Glucose tolerance

To determine whether gastric *H. pylori* infection modulates how the host initiates glucose homeostasis, we gave an intraperitoneal glucose challenge to experimental animals and evaluated the kinetics of plasma glucose from 0 to 90 minutes following glucose injection (Figure 3). In the db/db model, glucose levels fell toward normal levels more rapidly than in the *H. pylori* ^+^ mice (Panel 3A). In addition, the AUC calculations of GTT indicate that *H. pylori* ^+^ mice had lower levels than uninfected control mice (Panel 3C). In contrast, in the DIO model, levels were relatively similar (Panels 3B&D). The microarray results suggested that *H. pylori* infection modulated gastric PPAR γ pathway expression. To validate this prediction and to characterize potential interactions between *H. pylori* infection, high-fat feeding and PPAR γ expression we conducted a follow up study using wild-type and PPAR γ null mice fed low- or high-fat diets. Mice fed regular AIN-93G diets showed no differences between strains or genotypes (Figure 4A,B). Our data shows that glucose levels in wild-type mice fed high-fat diet, and infected with *H. pylori* 99–305 were more rapidly normalized than in the uninfected or *H. pylori* 98–325 groups (Figure 4C). Of note, our data also demonstrates that the beneficial effects of strain 99–305 on glucose homeostasis were impaired in PPAR γ null mice. Specifically, while wild-type mice challenged with strain 99–305 showed improved glucose normalization at 15, 45 and 60 min, PPAR γ null mice exhibited a significant change only 15 min after glucose challenge (Figure 4C and D).

### Effect of *H. pylori* infection and PPAR γ expression on WAT macrophage accumulation

To determine whether gastric *H. pylori* infection modulates the infiltration of immune cells in the WAT, we isolated stromal vascular cells and performed flow cytometric analysis of macrophage and T cell subsets. Our data demonstrates that the infiltration of F4/80+CD11b+ macrophages in WAT of wild-type mice fed high-fat diets infected with *H. pylori* strain 99–305 was significantly lower than in mice infected with *H. pylori* 98–325 or uninfected control mice (Figure 5A). Of note, the beneficial effect of *H. pylori* 99–305 was reversed in mice lacking PPAR γ in immune and epithelial cells (Figure 5B).

### Effect of *H. pylori* colonization on gastric PPAR γ-responsive gene expression in mice

PPAR γ responsive genes (CD36 and FABP4) in stomachs of mice were assayed by real-time quantitative RT-PCR. Wild type mice on a high-fat diet infected with the *H. pylori* 99–305 showed an increased expression of CD36 and FABP4, suggesting a higher PPAR γ activity when compared the uninfected and *H. pylori* 98–325 infected groups (Figure 6).

## Discussion

Recognition of the pathogenic role of *H. pylori* in the development of diseases of the distal stomach, such as gastric cancer and peptic ulceration [5], [6] highlights its intimate interaction with host tissues. As such, from an evolutionary view, exploration of *H. pylori* roles in human gastric endocrine and immune functions is warranted. *H. pylori* is the dominant member of the gastric microbiota [58], [59] and has co-evolved with our ancestors since before humans left Africa [4]. That most mammalian species studied have their own gastric *Helicobacter* species supports pre-human origins of gastric colonization by this genus. Accordingly, it might be expected that *H. pylori* plays roles as a commensal or symbiont in the regulation of gastric physiology [60]. Indeed, *H. pylori* affects gastric acid secretion [61] and modulates gastric acidity [62]. The endocrine function of the stomach also can be modulated by *H. pylori*; for example, gastric *H. pylori* colonization down-regulates ghrelin and somatostatin [29], [63] secretion, and stimulates that of leptin [64], [65] and gastrin [66].

The results of these studies provide *in vivo* evidence that gastric infection with a *cag* PAI-negative *H. pylori* strain, but not with an isogenic *cag* PAI-positive strain, ameliorates glucose tolerance possibly by activating PPAR γ, modulating appetite-controlling hormones and suppressing inflammation. *H. pylori*-colonized human hosts always harbor strains that lack the *cag PAI*, since *cag*-positive infections also involve *cag*-negative strains [58] and both undergo adaptation processes and differentially interact with the host [67]. For instance, variation in acid susceptibility by CagA status may contribute to the differential colonization of gastric sites. The host-specific equilibrium that has been observed between *cag*-positive and negative strains may facilitate metabolic and mucosal immune homeostasis by tightly controlling the balance of effector versus regulatory responses.

Obesity-related insulin-resistance is associated with low-grade chronic inflammation [42]. Macrophage-specific gene expression is up-regulated in WAT of db/db mice [42] and inflammatory macrophage subsets infiltrate WAT following high-fat feeding in mice [50]; these contribute to enhanced inflammatory signaling, thereby causing insulin resistance and impairing glucose tolerance [68]. Herein, we provide preliminary evidence that the *cag PAI* mutant, but not the wild-type *H. pylori* strain, is associated with enhanced influx of Treg cells into WAT during obesity, which is consistent with a predominance of anti-inflammatory responses. *H. pylori*-induced gastritis is associated with recruitment of Treg cells into the antral mucosa, suggesting a putative role for this CD4^+^ T cell subset in both the persistence of *H. pylori* infection [69], and in modulating systemic inflammation. Infection with a *cag*-positive strain may involve co-infection with *cag*-negative strains [70] with potentially opposing effects on the host, which might help neutralize inflammatory damage and disease burden through immunoregulation.

Our results also show that mice infected with either of the *H. pylori* strains tested had increased gastric-derived leptin without affecting WAT leptin expression. These data are consistent with results of a recent clinical study in humans showing elevated plasma leptin concentrations in *H. pylori*-colonized subjects [71]. Changes in leptin levels alone could not explain reduced obesity in the present study, as mice infected with the *cag PAI* mutant strain, but not the wild-type *H. pylori* strain, had significantly reduced body weights on days 42 and 48 compared to uninfected mice, whereas the effects on leptin were observed in mice infected with either strain. The db/db mice lack the leptin receptor long isoform (ObRb), but express fully functional short isoforms (i.e., ObRa, ObRe, ObRc, ObRd and ObRf) that contribute to its full spectrum of *in vivo* effects. ObRa is functionally active in the colons of db/db, but not ob/ob mice [72]. Thus, in this study gastric-derived leptin could contribute to *H. pylori* effects in metabolism, through the leptin receptor short isoforms in general, and ObRa in particular. However, the suppressive effects of leptin on feed intake would be abrogated due to the mutation in the ObRb receptor. In contrast, the C57BL/6 wild-type mice with DIO had a 4-fold increase in gastric leptin concentrations. In that model, leptin interacted with all isoforms of its receptor and we also observed improved obesity-related outcomes.

*H. pylori* infection improved fasting blood glucose levels in both mouse models of obesity and diabetes, although improvements were greater in mice infected with the *cag PAI* mutant strain. The differences in glucose tolerance observed between the wild-type European and the *cag* PAI mutant strain may be due to chronic gastric inflammation caused by infection with the *cag* ^+^ strain; through such inflammation the *cag* PAI-associated tissue responses may contribute to insulin resistance and counteract improvements attributable to changes in gastric-derived hormones. Interestingly, in a follow up study using wild-type and tissue-specific PPAR γ null mice, we demonstrated that the loss of PPAR γ in immune and epithelial cells impaired the ability of *H. pylori* infection to ameliorate glucose normalization during a GTT in mice fed high-fat diets. Moreover, infection with the *cag* PAI mutant *H. pylori* strain resulted in upregulation of gastric PPAR γ responsive genes (i.e., CD36 and FABP4), suggesting increased PPAR γ activation *in vivo*. These *in vivo* findings illustrate the compounded effect of *H. pylori* colonization on gastric mucosa epithelial and immune cells, all of which express PPAR γ, and therefore portray a comprehensive assessment of the effect of *H. pylori* infection on host response. Interestingly, two recent clinical reports suggest an association between PPAR γ and *H. pylori*-related gastric carcinoma [73], [74] thereby providing a molecular basis for the possible role of *H. pylori* in controlling gastric inflammation, carcinogenesis and metabolism.

Both the bacterial ecology of the stomach [59] and host physiology change in the absence of *H. pylori*, a bacterium that has co-evolved and co-adapted with humans and that is now disappearing in modern societies that are showing epidemic increases in obesity and diabetes. The present study demonstrates the importance of gastric *H. pylori* interactions in host control of body weight and glucose tolerance and suggests the importance of PPAR γ as a central regulator of host-bacterial interactions. These data suggest that colonization by *H. pylori* strains lacking the *cag PAI* could provide partial protection against some metabolic disorders. Thus, if this theory holds true, the disappearance of *H. pylori* in developed countries may be a contributing factor to the epidemics of obesity and diabetes. Future studies will examine the mechanisms by which specific *H. pylori* strains modulate regulatory and effector pathways in the gastric mucosa, and their correlation with improvements of chronic inflammatory diseases.

## Supporting Information

Table S1 Composition of the control and high saturated fat diets.
(DOC)

Table S2 Oligonucleotide sequences for quantitative real-time PCR.
(DOC)

## References

[1] Mane SP, Dominguez-Bello MG, Blaser MJ, Sobral BW, Hontecillas R, et al (2010) Host-interactive genes in Amerindian *Helicobacter pylori* diverge from their Old World homologs and mediate inflammatory responses. J Bacteriol 192: 3078–3092. 20400544 10.1128/JB.00063-10 PMC2901691

[2] Blaser MJ (2008) Disappearing microbiota: *Helicobacter pylori* protection against esophageal adenocarcinoma. Cancer Prev Res (Phila Pa) 1: 308–311. 10.1158/1940-6207.CAPR-08-0170 19138974

[3] Perez-Perez GI, Salomaa A, Kosunen TU, Daverman B, Rautelin H, et al (2002) Evidence that cagA(+) *Helicobacter pylori* strains are disappearing more rapidly than cagA(−) strains. Gut 50: 295–298. 11839704 10.1136/gut.50.3.295 PMC1773149

[4] Linz B, Balloux F, Moodley Y, Manica A, Liu H, et al (2007) An African origin for the intimate association between humans and *Helicobacter pylori* . Nature 445: 915–918. 17287725 10.1038/nature05562 PMC1847463

[5] Danesh J (1999) *Helicobacter pylori* infection and gastric cancer: systematic review of the epidemiological studies. Aliment Pharmacol Ther 13: 851–856. 10383517 10.1046/j.1365-2036.1999.00546.x

[6] Permin H, Andersen LP (2005) Inflammation, immunity, and vaccines for *Helicobacter* infection. Helicobacter 10 Suppl 121–25. 16178967 10.1111/j.1523-5378.2005.00337.x

[7] Vieth M, Masoud B, Meining A, Stolte M (2000) *Helicobacter pylori* infection: protection against Barrett's mucosa and neoplasia? Digestion 62: 225–231. 11070405 10.1159/000007820

[8] Vaezi MF, Falk GW, Peek RM, Vicari JJ, Goldblum JR, et al (2000) CagA-positive strains of *Helicobacter pylori* may protect against Barrett's esophagus. Am J Gastroenterol 95: 2206–2211. 11007219 10.1111/j.1572-0241.2000.02305.x

[9] Chow WH, Blaser MJ, Blot WJ, Gammon MD, Vaughan TL, et al (1998) An inverse relation between cagA+ strains of *Helicobacter pylori* infection and risk of esophageal and gastric cardia adenocarcinoma. Cancer Res 58: 588–590. 9485003

[10] Blaser MJ, Chen Y, Reibman J (2008) Does *Helicobacter pylori* protect against asthma and allergy? Gut. 10.1136/gut.2007.133462 PMC3888205 18194986

[11] Chen Y, Blaser MJ (2007) Inverse associations of *Helicobacter pylori* with asthma and allergy. Arch Intern Med 167: 821–827. 17452546 10.1001/archinte.167.8.821

[12] Lang L (2007) Childhood acquisition of *Helicobacter pylori* linked to reduced asthma and allergy risk. Gastroenterology 133: 6. 17631119 10.1053/j.gastro.2007.05.011

[13] McCune A, Lane A, Murray L, Harvey I, Nair P, et al (2003) Reduced risk of atopic disorders in adults with *Helicobacter pylori* infection. Eur J Gastroenterol Hepatol 15: 637–640. 12840675 10.1097/00042737-200306000-00010

[14] Codolo G, Mazzi P, Amedei A, Del Prete G, Berton G, et al (2008) The neutrophil-activating protein of *Helicobacter pylori* down-modulates Th2 inflammation in ovalbumin-induced allergic asthma. Cell Microbiol 10: 2355–2363. 18671823 10.1111/j.1462-5822.2008.01217.x

[15] Bourzac KM, Guillemin K (2005) *Helicobacter pylori*-host cell interactions mediated by type IV secretion. Cell Microbiol 7: 911–919. 15953024 10.1111/j.1462-5822.2005.00541.x

[16] Allen LA, Schlesinger LS, Kang B (2000) Virulent strains of *Helicobacter pylori* demonstrate delayed phagocytosis and stimulate homotypic phagosome fusion in macrophages. J Exp Med 191: 115–128. 10620610 10.1084/jem.191.1.115 PMC2195807

[17] Zheng PY, Jones NL (2003) *Helicobacter pylori* strains expressing the vacuolating cytotoxin interrupt phagosome maturation in macrophages by recruiting and retaining TACO (coronin 1) protein. Cell Microbiol 5: 25–40. 12542468 10.1046/j.1462-5822.2003.00250.x

[18] Rittig MG, Shaw B, Letley DP, Thomas RJ, Argent RH, et al (2003) *Helicobacter pylori*-induced homotypic phagosome fusion in human monocytes is independent of the bacterial vacA and cag status. Cell Microbiol 5: 887–899. 14641174 10.1046/j.1462-5822.2003.00328.x

[19] Kranzer K, Sollner L, Aigner M, Lehn N, Deml L, et al (2005) Impact of *Helicobacter pylori* virulence factors and compounds on activation and maturation of human dendritic cells. Infect Immun 73: 4180–4189. 15972508 10.1128/IAI.73.7.4180-4189.2005 PMC1168582

[20] Ohnishi N, Yuasa H, Tanaka S, Sawa H, Miura M, et al (2008) Transgenic expression of *Helicobacter pylori* CagA induces gastrointestinal and hematopoietic neoplasms in mouse. Proc Natl Acad Sci U S A 105: 1003–1008. 18192401 10.1073/pnas.0711183105 PMC2242726

[21] Peek RM Jr (2005) Orchestration of aberrant epithelial signaling by *Helicobacter pylori* CagA. Sci STKE 2005: pe14. 15798102 10.1126/stke.2772005pe14

[22] Selbach M, Moese S, Hauck CR, Meyer TF, Backert S (2002) Src is the kinase of the *Helicobacter pylori* CagA protein in vitro and in vivo. J Biol Chem 277: 6775–6778. 11788577 10.1074/jbc.C100754200

[23] Segal ED, Cha J, Lo J, Falkow S, Tompkins LS (1999) Altered states: involvement of phosphorylated CagA in the induction of host cellular growth changes by *Helicobacter pylori* . Proc Natl Acad Sci U S A 96: 14559–14564. 10588744 10.1073/pnas.96.25.14559 PMC24475

[24] Suzuki M, Mimuro H, Kiga K, Fukumatsu M, Ishijima N, et al (2009) *Helicobacter pylori* CagA phosphorylation-independent function in epithelial proliferation and inflammation. Cell Host Microbe 5: 23–34. 19154985 10.1016/j.chom.2008.11.010

[25] Lee IO, Kim JH, Choi YJ, Pillinger MH, Kim SY, et al (2010) *Helicobacter pylori* CagA phosphorylation status determines the gp130-activated SHP2/ERK and JAK/STAT signal transduction pathways in gastric epithelial cells. J Biol Chem 285: 16042–16050. 20348091 10.1074/jbc.M110.111054 PMC2871473

[26] Bagnoli F, Buti L, Tompkins L, Covacci A, Amieva MR (2005) *Helicobacter pylori* CagA induces a transition from polarized to invasive phenotypes in MDCK cells. Proc Natl Acad Sci U S A 102: 16339–16344. 16258069 10.1073/pnas.0502598102 PMC1274241

[27] Amieva MR, Vogelmann R, Covacci A, Tompkins LS, Nelson WJ, et al (2003) Disruption of the epithelial apical-junctional complex by *Helicobacter pylori* CagA. Science 300: 1430–1434. 12775840 10.1126/science.1081919 PMC3369828

[28] Shiotani A, Miyanishi T, Uedo N, Iishi H (2005) *Helicobacter pylori* infection is associated with reduced circulating ghrelin levels independent of body mass index. Helicobacter 10: 373–378. 16181346 10.1111/j.1523-5378.2005.00343.x

[29] Nwokolo CU, Freshwater DA, O'Hare P, Randeva HS (2003) Plasma ghrelin following cure of *Helicobacter pylori* . Gut 52: 637–640. 12692045 10.1136/gut.52.5.637 PMC1773634

[30] Liew PL, Lee WJ, Lee YC, Chen WY (2006) Gastric ghrelin expression associated with *Helicobacter pylori* infection and chronic gastritis in obese patients. Obes Surg 16: 612–619. 16687031 10.1381/096089206776945002

[31] Inui A, Asakawa A, Bowers CY, Mantovani G, Laviano A, et al (2004) Ghrelin, appetite, and gastric motility: the emerging role of the stomach as an endocrine organ. FASEB J 18: 439–456. 15003990 10.1096/fj.03-0641rev

[32] Antuna-Puente B, Feve B, Fellahi S, Bastard JP (2008) Adipokines: the missing link between insulin resistance and obesity. Diabetes Metab 34: 2–11. 18093861 10.1016/j.diabet.2007.09.004

[33] Lago R, Gomez R, Lago F, Gomez-Reino J, Gualillo O (2008) Leptin beyond body weight regulation–current concepts concerning its role in immune function and inflammation. Cell Immunol 252: 139–145. 18289518 10.1016/j.cellimm.2007.09.004

[34] Kalra SP, Ueno N, Kalra PS (2005) Stimulation of appetite by ghrelin is regulated by leptin restraint: peripheral and central sites of action. J Nutr 135: 1331–1335. 15867335 10.1093/jn/135.5.1331

[35] Jequier E (2002) Leptin signaling, adiposity, and energy balance. Ann N Y Acad Sci 967: 379–388. 12079865 10.1111/j.1749-6632.2002.tb04293.x

[36] Hossain P, Kawar B, El Nahas M (2007) Obesity and diabetes in the developing world–a growing challenge. N Engl J Med 356: 213–215. 17229948 10.1056/NEJMp068177

[37] CDC (2005) National Diabetes Fact Sheet: general information and national estimates on diabetes in the United States. Atlanta, GA. 1–10 p.

[38] Kamada T, Hata J, Kusunoki H, Ito M, Tanaka S, et al (2005) Eradication of *Helicobacter pylori* increases the incidence of hyperlipidaemia and obesity in peptic ulcer patients. Dig Liver Dis 37: 39–43. 15702858 10.1016/j.dld.2004.07.017

[39] Cover TL, Blaser MJ (2009) *Helicobacter pylori* in health and disease. Gastroenterology 136: 1863–1873. 19457415 10.1053/j.gastro.2009.01.073 PMC3644425

[40] Wu MS, Lee WJ, Wang HH, Huang SP, Lin JT (2005) A case-control study of association of *Helicobacter pylori* infection with morbid obesity in Taiwan. Arch Intern Med 165: 1552–1555. 16009873 10.1001/archinte.165.13.1552

[41] Shoelson SE, Lee J, Goldfine AB (2006) Inflammation and insulin resistance. J Clin Invest 116: 1793–1801. 16823477 10.1172/JCI29069 PMC1483173

[42] Xu H, Barnes GT, Yang Q, Tan G, Yang D, et al (2003) Chronic inflammation in fat plays a crucial role in the development of obesity-related insulin resistance. J Clin Invest 112: 1821–1830. 14679177 10.1172/JCI19451 PMC296998

[43] Weisberg SP, McCann D, Desai M, Rosenbaum M, Leibel RL, et al (2003) Obesity is associated with macrophage accumulation in adipose tissue. J Clin Invest 112: 1796–1808. 14679176 10.1172/JCI19246 PMC296995

[44] Tebbutt NC, Giraud AS, Inglese M, Jenkins B, Waring P, et al (2002) Reciprocal regulation of gastrointestinal homeostasis by SHP2 and STAT-mediated trefoil gene activation in gp130 mutant mice. Nat Med 8: 1089–1097. 12219085 10.1038/nm763

[45] Wang TC, Goldenring JR (2002) Inflammation intersection: gp130 balances gut irritation and stomach cancer. Nat Med 8: 1080–1082. 12357240 10.1038/nm1002-1080

[46] Allen LA (2007) Phagocytosis and persistence of *Helicobacter pylori* . Cell Microbiol 9: 817–828. 17346311 10.1111/j.1462-5822.2007.00906.x

[47] Menaker RJ, Ceponis PJ, Jones NL (2004) *Helicobacter pylori* induces apoptosis of macrophages in association with alterations in the mitochondrial pathway. Infect Immun 72: 2889–2898. 15102801 10.1128/IAI.72.5.2889-2898.2004 PMC387848

[48] D'Elios MM, Codolo G, Amedei A, Mazzi P, Berton G, et al (2009) *Helicobacter pylori*, asthma and allergy. FEMS Immunol Med Microbiol 56: 1–8. 19220467 10.1111/j.1574-695X.2009.00537.x

[49] Myers MG Jr (2004) Leptin receptor signaling and the regulation of mammalian physiology. Recent Prog Horm Res 59: 287–304. 14749507 10.1210/rp.59.1.287

[50] Bassaganya-Riera J, Misyak S, Guri AJ, Hontecillas R (2009) *PPAR gamma* is highly expressed in F4/80(hi) adipose tissue macrophages and dampens adipose-tissue inflammation. Cell Immunol 258: 138–146. 19423085 10.1016/j.cellimm.2009.04.003 PMC2706276

[51] Akopyants NS, Clifton SW, Kersulyte D, Crabtree JE, Youree BE, et al (1998) Analyses of the cag pathogenicity island of *Helicobacter pylori* . Mol Microbiol 28: 37–53. 9593295 10.1046/j.1365-2958.1998.00770.x

[52] Guri AJ, Hontecillas R, Ferrer G, Casagran O, Wankhade U, et al (2008) Loss of *PPAR gamma* in immune cells impairs the ability of abscisic acid to improve insulin sensitivity by suppressing monocyte chemoattractant protein-1 expression and macrophage infiltration into white adipose tissue. J Nutr Biochem 19: 216–228. 17618105 10.1016/j.jnutbio.2007.02.010

[53] Bassaganya-Riera J, Reynolds K, Martino-Catt S, Cui Y, Hennighausen L, et al (2004) Activation of PPAR gamma and delta by conjugated linoleic acid mediates protection from experimental inflammatory bowel disease. Gastroenterology 127: 777–791. 15362034 10.1053/j.gastro.2004.06.049

[54] SAS (1988) SAS/STAT User's guide (Release 6.0.3). Cary, NC: SAS Inst. Inc.

[55] Hontecillas R, Wannemeulher MJ, Zimmerman DR, Hutto DL, Wilson JH, et al (2002) Nutritional regulation of porcine bacterial-induced colitis by conjugated linoleic acid. J Nutr 132: 2019–2027. 12097686 10.1093/jn/132.7.2019

[56] Bassaganya-Riera J, Pogranichniy RM, Jobgen SC, Halbur PG, Yoon KJ, et al (2003) Conjugated Linoleic Acid Ameliorates Viral Infectivity in a Pig Model of Virally Induced Immunosuppression. J Nutr 133: 3204–3214. 14519812 10.1093/jn/133.10.3204

[57] Matarese G, Procaccini C, De Rosa V, Horvath TL, La Cava A (2010) Regulatory T cells in obesity: the leptin connection. Trends Mol Med. 10.1016/j.molmed.2010.04.002 20493774

[58] Ghose C, Perez-Perez GI, van Doorn LJ, Dominguez-Bello MG, Blaser MJ (2005) High frequency of gastric colonization with multiple *Helicobacter pylori* strains in Venezuelan subjects. J Clin Microbiol 43: 2635–2641. 15956377 10.1128/JCM.43.6.2635-2641.2005 PMC1151950

[59] Bik EM, Eckburg PB, Gill SR, Nelson KE, Purdom EA, et al (2006) Molecular analysis of the bacterial microbiota in the human stomach. Proc Natl Acad Sci U S A 103: 732–737. 16407106 10.1073/pnas.0506655103 PMC1334644

[60] Atherton JC, Blaser MJ (2009) Coadaptation of *Helicobacter pylori* and humans: ancient history, modern implications. J Clin Invest 119: 2475–2487. 19729845 10.1172/JCI38605 PMC2735910

[61] McColl KE, el-Omar E, Gillen D (2000) *Helicobacter pylori* gastritis and gastric physiology. Gastroenterol Clin North Am 29: 687–703, viii. 10.1016/s0889-8553(05)70138-2 11030081

[62] Tsai SH, Chen CM, Chang CS, Chen GH (2004) Effect of *Helicobacter pylori* infection on intragastric acidity in patients with reflux esophagitis. J Gastroenterol 39: 821–826. 15565399 10.1007/s00535-004-1396-8

[63] Shiotani A, Miyanishi T, Uedo N, Iishi H (2005) *Helicobacter pylori* infection is associated with reduced circulating ghrelin levels independent of body mass index. Helicobacter 10: 373–378. 16181346 10.1111/j.1523-5378.2005.00343.x

[64] Roper J, Francois F, Shue PL, Mourad MS, Zhiheng P, et al. (2007) Leptin and ghrelin in relation to *Helicobacter pylori* status in adult males. Journal of Clinical Endocrinology & Metabolism 10 2007–2057. 10.1210/jc.2007-2057 PMC2435636 18397989

[65] Azuma T, Suto H, Ito Y, Ohtani M, Dojo M, et al (2001) Gastric leptin and *Helicobacter pylori* infection. Gut 49: 324–329. 11511551 10.1136/gut.49.3.324 PMC1728440

[66] Kaneko H, Konagaya T, Kusugami K (2002) *Helicobacter pylori* and gut hormones. J Gastroenterol 37: 77–86. 11871770 10.1007/s005350200000

[67] Karita M, Blaser MJ (1998) Acid-tolerance response in *Helicobacter pylori* and differences between cagA+ and cagA-strains. J Infect Dis 178: 213–219. 9652443 10.1086/515606

[68] Wellen KE, Hotamisligil GS (2005) Inflammation, stress, and diabetes. J Clin Invest 115: 1111–1119. 15864338 10.1172/JCI25102 PMC1087185

[69] Kandulski A, Wex T, Kuester D, Peitz U, Gebert I, et al (2008) Naturally occurring regulatory T cells (CD4+, CD25high, FOXP3+) in the antrum and cardia are associated with higher *Helicobacter pylori* colonization and increased gene expression of TGF-beta1. Helicobacter 13: 295–303. 18665940 10.1111/j.1523-5378.2008.00612.x

[70] Secka O, Antonio M, Berg DE, Tapgun M, Bottomley C, et al (2011) Mixed infection with cagA positive and cagA negative strains of *Helicobacter pylori* lowers disease burden in The Gambia. PLoS One 6: e27954. 22140492 10.1371/journal.pone.0027954 PMC3226634

[71] Konturek PC, Czesnikiewicz-Guzik M, Bielanski W, Konturek SJ (2006) Involvement of *Helicobacter pylori* infection in neuro-hormonal control of food intake. J Physiol Pharmacol 57 Suppl 567–81. 17218760

[72] Ealey KN, Lu S, Archer MC (2008) Development of aberrant crypt foci in the colons of ob/ob and db/db mice: evidence that leptin is not a promoter. Mol Carcinog 47: 667–677. 18240295 10.1002/mc.20419

[73] Bazargani A, Khoramrooz SS, Kamali-Sarvestani E, Taghavi SA, Saberifiroozi M (2010) Association between *peroxisome proliferator-activated receptor-gamma* gene polymorphism (Pro12Ala) and *Helicobacter pylori* infection in gastric carcinogenesis. Scand J Gastroenterol 45: 1162–1167. 20568969 10.3109/00365521.2010.499959

[74] Yao L, Liu F, Sun L, Wu H, Guo C, et al (2010) Upregulation of *PPARgamma* in tissue with gastric carcinoma. Hybridoma (Larchmt) 29: 341–343. 20715992 10.1089/hyb.2010.0013

