# Supplementary material for: Helicobacter pylori Colonization Ameliorates Glucose Homeostasis in Mice through a PPAR γ-Dependent Mechanism
Source: PLoS One. 2012 Nov 15;7(11):e50069. doi: 10.1371/journal.pone.0050069 (PMC3499487; doi:10.1371/journal.pone.0050069)
Supplement: Table S1 — Composition of the control and high saturated fat diets. (DOC) [file pone.0050069.s001.doc]

**Table S1. Composition of the control and high saturated fat diets.**

| **Ingredient** (g/kg) | Control Diet | High Fat Diet ^1^ |
| --- | --- | --- |
| Casein | 200 | 232 |
| L-Cystine | 3.0 | 3.0 |
| DL-Methionine | -- | 3.5 |
| Corn Starch | 397.48 | 137 |
| Maltodextrin | 132 | 150 |
| Sucrose | 100 | 162.58 |
| Cellulose | 50 | 50 |
| Cholesterol | -- | 1.9 |
| Mineral Mix (AIN-93)^2^ | 35 | 40.60 |
| Calcium phosphate dibasic | -- | 4.64 |
| Vitamin Mix (AIN-93)^3^ | 10 | 16.24 |
| Choline Bitartrate^4^ | 2.5 | 5 |
| tert-butylhydroquinone^5^ | 0.014 | 0.04 |
| Soybean oil | 70 | 40 |
| Lard | -- | 153.5 |

^1^ Provides approximately 19.6% fat, 0.2% total cholesterol and 4.4 kilocalories/g (kcal/g) providing 40% kcal from fat. Kcal density is approximately 16% higher than typical AIN-93G-based diets.

^2^ Supplied per kg of vitamin mix: 3 g nicotinic acid, 1.6 g calcium pantotenate, 0.7 g pyridoxine HCl, 0.6 g Thiamin HCl, 0.6 g riboflavin, 0.2 g folic acid, 0.02 g D-biotin, 2.5 g vitamin B_12_ (0.1 % in mannitol), 15 g DL-alpha tocopheryl acetate (500 IU/g), 0.8 g vitamin A palmitate (500,000 IU/g), 0.2 g vitamin D_3_ (cholecalciferol, 500,000 IU/g), 0.075 g vitamin K (phylloquinone), and 974.705 g sucrose.

^3^ Supplied per kg of mineral mix: 357 g calcium carbonate, 196 g potassium phosphate monobasic, 70.78 g potassium citrate, 74 g sodium chloride, 46.6 g potassium sulfate, 24.3 g magnesium oxide, 6.06 g ferric citrate, 1.65 g zinc carbonate, 0.63 g manganous carbonate, 0.31 g cupric carbonate, 0.01 g potassium iodate, 0.01025 g sodium selenate, 0.00795 g ammonium paramolybdate, 1.45 g sodium meta-silicate, 0.275 g chromium potassium sulfate, 0.0174 g lithium chloride, 0.0815 g boric acid, 0.0635 g sodium fluoride, 0.0318 g nickel carbonate, hydroxide, tetrahydrate, 0.0066 g ammonium vanadate, and 220.716 g sucrose.

^4^ The choline bitartrate concentrations have been increased from 2.5 g/kg in regular AIN-93G diets to 5 g/kg due to increased kcal density of high fat diets.

^5^ Antioxidant.
