# Supplementary material for: Helicobacter pylori Colonization Ameliorates Glucose Homeostasis in Mice through a PPAR γ-Dependent Mechanism
Source: PLoS One. 2012 Nov 15;7(11):e50069. doi: 10.1371/journal.pone.0050069 (PMC3499487; doi:10.1371/journal.pone.0050069)
Supplement: Table S2 — Oligonucleotide sequences for quantitative real-time PCR. (DOC) [file pone.0050069.s002.doc]

| **Table S2.** Oligonucleotide sequences for quantitative real-time PCR. ^a, b^ | | | | | |
| --- | --- | --- | --- | --- | --- |
| Primer | Sequence | Length | | | Accession Number |
| β-actinF | 5′CCCAGGCATTGCTGACAGG3′ | | | 141 | X03672 |
| β-actinR | 5′TGGAAGGTGGACAGTGAGGC3′ | | |  |  |
| LeptinF | 5′GATCTCACAATGCGTTTCTT3′ | | | 158 | BC125245 |
| LeptinR | 5′GCTCAGGACCATCTGCTA3′ | | |  |  |
| GhrelinF | 5′GGCCCTGGGGAAGTTTCTT3′ | | | 130 | AB035701 |
| GhrelinR | 5′GCTGAGGCGGATGTGAGTTC3′ | | |  |  |
| IL-6F | 5′TCAATTCCAGAAACCGCTATG3′ | | | 120 | NM_031168 |
| Il-6R | 5′TCCGGACTTGTGAAGTAGGG3′ | | |  |  |
| CD36F | 5'-CCGGGCCACGTAGAAAACA-3' | |  | 156 | NM_007643 |
| CD36R | 5'-CCTCCAAACACAGCCAGGAC-3' | |  |  |  |
| FABP4F | 5'-TGTGTTATGAAAGGCGTGACTTCC-3' | |  | 78 | NM_024406 |
| FABP4R | 5'-CAAATTTCCATCCAGGCCTCTTC-3' | |  |  |  |

**^a^** F, forward; R, reverse. PCR primer pairs were designed for amplicon lengths ranging between 73 and 157 base pairs. Annealing temperatures are 57ºC for β-actin, CD36, FABP4. For ghrelin, 54 ºC for IL-6 and 52.2ºC for leptin.

^b^ When plotting threshold cycle versus log starting quantity (pg), standard curves had slopes between -3.1 and -3.7; PCR efficiencies above 92% and R^2^ above 0.98.
